# 中国首次复发多发性骨髓瘤诊治指南（2022年版）

**DOI:** 10.3760/cma.j.issn.0253-2727.2022.10.003

**Published:** 2022-10

**Authors:** 中华医学会血液学分会浆细胞疾病学组 中国医师协会多发性骨髓瘤专业委员会

一、概述

随着自体造血干细胞移植（autologous hematopoietic stem cell transplantation，ASCT）和新药在一线治疗中的应用，多发性骨髓瘤（multiple myeloma，MM）患者的总生存（overall survival，OS）期已明显延长，但MM仍是一种不可治愈的疾病[1]–[2]，患者终将面临复发。复发按照发生次数分为首次复发（第一次复发）和多线复发（第二次或以上复发），多线复发的患者获得高危细胞遗传学的概率增加，治疗难度随之增加，复发次数越多，无进展生存（progression-free survival，PFS）和复发后的生存时间越短[3]–[4]。部分患者因各种原因可能仅有两次机会接受抗骨髓瘤治疗[5]–[6]。因此，首次复发MM患者的治疗非常重要，恰当的治疗可使这部分患者最大程度获益。随着MM新药的增加，临床医师在首次复发患者的治疗选择上有很多困惑和较大差异，我国目前尚无针对首次复发MM诊治的专家共识或指南，与欧美国家相比，我国新药的可及性仍相对有限。如何利用有限的新药和治疗手段让患者最大程度获益是临床迫切需要解决的问题，因此，有必要制定适合我国首次复发MM的诊治指南。

二、首次复发的定义

由于复发的表现形式多样，目前国内外MM疗效判定标准中没有对复发进行统一定义，根据复发的形式常用以下指标：临床复发、生化复发、完全缓解（complete remission，CR）后复发、微小残留病（minimal residual disease，MRD）由阴转阳的复发[1],[7]–[10]。MM患者在接受一线治疗期间或治疗后曾达到过疾病缓解，出现以上情况之一时则定义为首次复发。

1. 临床复发：符合以下一项或多项：①出现新的骨病变（骨质疏松性骨折除外）或软组织浆细胞瘤；②明确的（可测量病变最大垂直径乘积之和增加50％且绝对值≥1 cm）已有的浆细胞瘤或骨病变增加；③高钙血症（校正血清钙>2.75 mmol/L）；④血红蛋白浓度下降≥20 g/L（与治疗或非MM因素无关）；⑤从MM治疗开始，血肌酐上升≥176.8 µmol/L（2 mg/dl）且与MM相关；⑥血清M蛋白相关的高黏滞血症。

2. 生化复发：符合以下条件中任意一项，且无MM相关器官功能损害或症状定义为生化复发：①血清M蛋白升高≥25％（升高绝对值需≥5 g/L）；②尿M蛋白升高≥25％（升高绝对值需≥200 mg/24 h）；③若血清和尿M蛋白无法检出，受累与非受累血清游离轻链（free light chain，FLC）差值增加≥25％（增加绝对值需>100 mg/L）；④骨髓浆细胞比例增加绝对值≥10％。

3. CR后复发：免疫固定电泳证实血或尿M蛋白再次出现或骨髓浆细胞比例≥5％。

4. MRD阴性后复发：连续监测失去MRD阴性状态［二代流式细胞术（NGF）或二代测序（NGS）证实存在克隆性浆细胞，或影像学提示MM复发］。MRD敏感性规定至少在10^−5^水平，建议应用NGF或NGS进行检测。影像学建议应用全身PET-CT评估。

特殊形式复发：部分患者以髓外浆细胞瘤的形式复发，定义见“1. 临床复发”的“①”和“②”；也有部分MM患者以浆细胞白血病的形式复发（继发性浆细胞白血病），表现为外周血浆细胞比例≥20％和（或）外周血浆细胞绝对值≥2×10^9^/L。

根据复发的生物学特征可分为侵袭性复发和非侵袭性复发，具备下列任意一项者定义为侵袭性复发：①新出现不良的细胞遗传学异常，如：t（4;14）、17p−、1q21+和亚二倍体；②高β_2_微球蛋白（≥5.5 mg/L）或低白蛋白（<35 g/L）；③出现髓外浆细胞瘤；④LDH（与MM相关）大于正常值上限；⑤缓解期短或治疗中出现疾病进展；⑥出现侵袭性临床表现，包括快速出现症状，实验室、影像学或病理检查发现广泛的疾病进展、疾病相关的器官功能不全；⑦外周血出现浆细胞；⑧复发时ISS分期Ⅱ/Ⅲ期；⑨免疫球蛋白类型转化（轻链逃逸，浆细胞分泌活性降低）[1]。

三、首次复发需要完善的检查

如考虑复发，建议完善表1中所列项目。其中，细胞遗传学和全身PET-CT非常重要，即使新诊断时存在高危细胞遗传学异常，复发后仍需再次评估，以确定是否获得更高比例的相同高危细胞遗传学异常或增加预后差的细胞遗传学异常（如双打击、三打击等），全身PET-CT主要用于评估髓外病变，建议有条件者增加MM相关基因突变检查。

**表1:** 多发性骨髓瘤首次复发需要完善的检查

项目	内容
血液检查	血常规、肝肾功能（包括肌酐、白蛋白、球蛋白、乳酸脱氢酶、碱性磷酸酶、尿酸）、电解质（包括钙离子）、凝血功能、β_2_微球蛋白、外周血涂片（浆细胞比例）；血M蛋白相关检查：血清免疫球蛋白定量、血清蛋白电泳、血免疫固定电泳、血清游离轻链
尿液检查	尿常规、24 h尿蛋白定量、24 h尿轻链检测、尿蛋白电泳、尿免疫固定电泳
骨髓检查	骨髓细胞涂片±活检（浆细胞比例）；流式细胞术检查：CD38/CD138/CD56/CD19/CD20/cκ/cλ；荧光原位杂交（FISH）：包括但不限于以下内容：17p缺失、13q14缺失、1q21扩增、1p32缺失、t（4;14）、t（6;14）、t（11;14）、t（14;16）、t(14;20)；有条件者行相关基因突变的测定
影像学检查	全身X线平片（包括扁骨和长骨）；全身PET-CT；如无法行全身PET-CT，建议行MRI或全身低剂量CT
其他（患者器官功能的评估）	心电图、氨基末端脑钠肽前体（NT-proBNP）、肌钙蛋白T（TnT）、超声心动图、胸部CT、腹部B超等，必要时肺功能检测

四、首次复发治疗选择需考虑的因素

1. 患者一般状况的评估：复发时的年龄、合并疾病（如糖尿病、高血压、冠心病、慢性阻塞性肺病等）、体能状态（ECOG评分）或虚弱评分（推荐IMWG GA评分）[11]、经济状况、个人意愿。重要脏器功能评估如心脏、肺功能、肝功能、肾功能等。

2. 复发后疾病状态的评估：需要评估MM复发的形式和特征（临床复发、生化复发、CR后复发、MRD由阴转阳复发、是否为侵袭性复发等），全身PET-CT有无合并髓外病变，细胞遗传学评估复发后的获得性改变等。

3. 既往一线治疗情况的评估：

（1）一线治疗的方式和药物：一线治疗是否行ASCT，一线治疗应用药物情况，如蛋白酶体抑制剂（PIs）、免疫调节剂（IMiDs）、达雷妥尤单抗（Dara）等，充分评估患者在一线治疗期间药物暴露和可能存在的耐药或难治情况。

（2）一线治疗缓解深度及持续时间：需评估一线治疗获得的最佳疗效及疗效持续时间，复发时与第一次ASCT的间隔时间，距离首次有效诱导方案治疗的时间等，这对于选择首次复发的治疗方案非常重要。

（3）一线治疗相关毒性：如一线治疗出现硼替佐米相关的1级伴疼痛或2级以上周围神经炎，硼替佐米不再推荐继续应用；对于发生过深静脉血栓的患者，尽可能避免再次应用含免疫调节剂的方案，如果确需继续使用，建议给予标准的预防血栓治疗。

五、首次复发的治疗选择

（一）首次复发的治疗目标和治疗时机选择

1. 治疗目标：首次复发的治疗目标是获得最大程度的缓解，延长PFS时间。

2. 治疗时机的选择：不是所有MM患者在复发后即需启动治疗，应根据不同的复发形式和复发特征作出选择。如果为临床复发，重新出现CRAB现象或髓外病变或浆细胞白血病，则需即刻启动治疗。对于生化复发的患者，如果单克隆免疫球蛋白的增长速度符合侵袭性复发的标准，建议尽早启动治疗。对于M蛋白增长缓慢的生化复发患者，建议可先观察，每2～3个月评估1次，但也有临床试验证实此阶段早期干预可使患者获益。MRD由阴性转为阳性的复发是否需要干预尚不明确，有研究显示MRD阴性转阳性的患者临床复发风险显著升高，且与较差的PFS和OS相关[12]–[13]，目前已有把MRD阴性复发作为MM治疗指征的临床研究[14]。中山大学附属第一医院单中心资料显示，MRD阴性转阳性时异常浆细胞占总浆细胞比例>51％且伴高危细胞遗传学的患者，2个月内出现生化和临床复发的概率明显增加，对于这些患者可能需要提前干预[15]。因此，目前对于MRD由阴性转阳性患者的处理以观察为主，也可开展临床研究。

（二）我国首次复发MM可选择的药物和治疗方法

表2列举了国际上多个关键的Ⅲ期临床研究，首次复发患者采用不同治疗方案的疗效供临床参考，目前我国首次复发MM有如下四种治疗选择：

1. 免疫治疗：免疫治疗包括单克隆抗体（mAb）、双特异性抗体（BsAb）、抗体偶联细胞毒药物（ADC）、嵌合抗原受体T（CAR-T）细胞治疗等。BsAb和ADC在我国尚未获批，因此暂不推荐用于首次复发的患者。CAR-T细胞治疗虽在我国MM领域取得很大成绩，但考虑其尚未在我国正式获批，建议患者可以参加临床研究。

目前我国获批的单克隆抗体为达雷妥尤单抗，两个大型Ⅲ期临床研究即POLLUX和CASTOR试验奠定了达雷妥尤单抗联合Rd或Vd在复发难治MM（RRMM）中的地位，含达雷妥尤单抗的方案起效快，可获得更高的总反应率（ORR）和MRD阴性率，并转化为更长的PFS时间和OS时间，尤其在首次复发患者中疗效显著[16]–[19]。国内开展的LEPUS临床研究结果与CASTOR结果相似[20]。CANDOR和APOLLO临床试验是达雷妥尤单抗分别联合Kd或Pd，在首次复发MM患者中的疗效均较对照组（Kd或Pd）好[21]–[23]。单克隆抗体的不良反应主要为输注相关反应（IRR）、感染、中性粒细胞减少和血小板减少症等，皮下注射剂型可以明显减轻IRR的发生。

**表2:** 首次复发多发性骨髓瘤关键Ⅲ期临床研究

研究名称	方案	例数（例）	首次复发率	疗效	PFS	OS
POLLUX[16]–[17]	DRd对Rd	286对283	52%对52%	首次复发：ORR：93%对80%，≥CR率：59%对29%，MRD阴性率：32%对10%	首次复发：53.5个月对19.6个月（*HR*=0.42）	首次复发：77.8个月对57.7个月（*HR*=0.75）
CASTOR[18]–[19]	DVd对Vd	251对247	49%对46%	首次复发：ORR：92%对74%，≥CR率：43%对15%，MRD阴性率：21%对3%	首次复发：27.0个月对7.9个月（*HR*=0.21）	首次复发：未达到对47.0个月（*HR*=0.56）
LEPUS（中国）[20]	DVd对Vd	141对70	29%对27%	ORR：83%对65%，≥CR率：33%对11%，MRD阴性率：22%对3%	未达到对6.3个月（*HR*=0.16）	12个月OS率：88%对68%
CANDOR[21]–[22]	DKd对Kd	312对154	46%对45%	首次复发：ORR：91%对76%，≥CR率：41%对13%，MRD阴性率：17%对2%	首次复发：未达到对21.3个月（*HR*=0.66）	18个月OS率：80%对74%
APOLLO[23]	DPd对Pd	151对153	11%对12%	ORR：69%对46%，≥CR率：25%对4%，MRD阴性率：9%对2%	首次复发：14.1个月对12.6个月（*HR*=0.70）	未达到
ASPIRE[24]	KRd对Rd	396对396	46%对40%	首次复发：ORR：87.0%对70.1%，≥CR率：33.7%对7%，sCR率：12.5%对3.2%	首次复发：29.6个月对17.6个月（*HR*=0.69）	首次复发：47.3个月对35.9个月（*HR*=0.81）
IKEMA-MM[25]	Isa-Kd对Kd	179对123	44%对45%	ORR：87.5%对85.5%，CR率：41.3%对36.4%，MRD阴性率：33.8%对18.2%	首次复发：18个月PFS率：68%对45%	未达到
ENDEAVOR[26]	Kd对Vd	464对465	50%对50%	首次复发：ORR：81.9%对70.1%，≥CR率：11.7%对7.8%，≥VGPR率：62.1%对30.6%	首次复发：22.1个月对10.1个月（*HR*=0.45）	首次复发：51.3个月对43.7个月（*HR*=0.77）
TOURMALINE-MM1[27]	IRd对Rd	360对362	62%对60%	ORR：78%对72%，≥CR率：12%对7%，≥VGPR率：48%对39%	首次复发：20.6个月对15.9个月（*HR*=0.83）	53.6个月对51.6个月（*HR*=0.94）
OPTIMISMM[28]	PVd对Vd	281对278	40%对41%	首次复发：ORR：90.1%对54.8%	首次复发：20.7个月对11.6个月（*HR*=0.54）	未达到
BOSTON[29]	SVd对Vd	195对207	50%对48%	首次复发：ORR：80.8%对65.7%	首次复发：16.6个月对10.6个月（*HR*=0.63）	未达到对25个月
BELLINI[30]	VenVd对Vd	194对97	47%对45%	ORR：82%对68%，≥CR率：26%对5%	首次复发：22.4个月对11.4个月（*HR*=0.75）	未达到

注：PFS：无进展生存；OS：总生存；ORR：总反应率；CR：完全缓解；sCR：严格意义的完全缓解；MRD：微小残留病；VGPR：非常好的部分缓解；DRd：达雷妥尤单抗+来那度胺+地塞米松；Rd：来那度胺+地塞米松；DVd：达雷妥尤单抗+硼替佐米+地塞米松；Vd：硼替佐米+地塞米松；DKd：达雷妥尤单抗+卡非佐米+地塞米松；Kd：卡非佐米+地塞米松；DPd：达雷妥尤单抗+泊马度胺+地塞米松；Pd：泊马度胺+地塞米松；KRd：卡非佐米+来那度胺+地塞米松；Rd：来那度胺+地塞米松；Isa-Kd：Isatuximab+卡非佐米+地塞米松；IRd：伊沙佐米+来那度胺+地塞米松；PVd：泊马度胺+硼替佐米+地塞米松；SVd：塞利尼索+硼替佐米+地塞米松；VenVd：维奈克拉+硼替佐米+地塞米松

2. 更换不同作用机制的药物或新一代PIs和（或）IMiDs：以PIs（主要是硼替佐米）为主的联合方案或以IMiDs［主要是沙利度胺和（或）来那度胺］为主的方案，或PIs+IMiDs联合用于MM的一线治疗已日渐普遍。如果一线治疗仅包含PIs，首次复发可选择IMiDs；如果一线治疗仅应用IMiDs，首次复发可选择PIs。也可选择相应的新一代IMiDs如泊马度胺及新一代PIs如卡非佐米。

（1）新一代IMiDs：泊马度胺是第三代IMiDs，在我国已批准用于RRMM的治疗。泊马度胺对于来那度胺耐药患者疗效显著，即使对来那度胺和硼替佐米双耐药的患者也有一定效果。OPTIMISMM研究是比较泊马度胺联合硼替佐米/地塞米松方案（PVd）和硼替佐米/地塞米松方案治疗RRMM患者的一项Ⅲ期随机对照试验（RCT），结果显示在首次复发MM患者中，PVd组的ORR、PFS明显优于对照组[28]。在我国真实世界研究中，Pd与达雷妥尤单抗组合的疗效优于Pd与其他药物组合。

（2）新一代PIs：卡非佐米是新一代PIs，目前已在我国获批用于RRMM患者的治疗。ASPIRE研究对比KRd和Rd在RRMM患者中的疗效，两组首次复发患者的例数分别为184例（46.5％）和157例（39.6％），结果显示KRd较Rd显著改善PFS和OS，其中首次复发患者的PFS获益更显著[25]。与硼替佐米相比，卡非佐米的周围神经炎发生率低，但需注意其对心血管系统的毒副作用。

伊沙佐米是一种口服的PIs，与硼替佐米相比，用药方便是其优势，但由于其起效相对缓慢，可用于非侵袭性复发的患者。

3. 其他作用机制的药物：其他作用机制的药物目前也在不断涌现，如核输出蛋白抑制剂塞利尼索（Selinexor）、Bcl-2抑制剂维奈克拉（Venetoclax）、烷化剂与靶向细胞内氨肽酶的多肽偶联物Melflufen等新药，但维奈克拉和Melflufen尚未在我国获批，暂不推荐用于首次复发的MM患者，除非进入临床试验。多种分子标记驱动的新药已在临床试验中，目前也暂不推荐用于首次复发MM患者的治疗。

塞利尼索可口服用药，在我国已批准用于治疗RRMM。BOSTON研究对比SVd和Vd在既往接受过1～3线治疗的MM患者中的疗效，两组首次复发的患者例数分别为99例（50.8％）和99例（47.8％），SVd和Vd应用于首次复发MM患者的ORR分别为81％和66％，≥VGPR率分别为51％和29.3％，SVd应用于首次复发MM患者的中位PFS时间明显优于Vd（16.6个月对10.6个月），疾病进展或死亡风险降低了37％[29]。

4. ASCT：一线治疗无论是否选择ASCT，MM患者在疾病复发后仍可再次选择ASCT[31]–[34]，复发后的ASCT分为以下两种情况。

（1）一线治疗时未采用ASCT的患者。目前有部分指南或临床试验推荐适合移植的标危MM患者在初始诱导后先采集造血干细胞冻存，但当时并不进行ASCT，而是按照不移植的流程完成后续治疗，待疾病复发后再选择ASCT，即晚期ASCT。IFM2009研究比较了一线ASCT和晚期移植的疗效，接近8年的随访结果显示两组的PFS和OS并无差异，说明晚期移植对于一线未接受ASCT的患者来说仍是首次复发的选择之一，但有接近25％一线不移植的患者由于各种原因会失去晚期移植的机会[35]–[36]，导致OS和PFS下降。由于各种因素，我国MM患者目前一线ASCT比例低，首次复发时如果患者一般状态和器官功能符合移植条件，仍可以考虑进行晚期ASCT。考虑到一线治疗时多次化疗可能会导致造血干细胞采集不足，对于移植候选者，即使一线暂不采用ASCT，建议在初始诱导4个疗程时先采集造血干细胞冻存备用。

（2）针对一线已经实施ASCT的患者，首次复发后可再进行第二次ASCT。目前有两项随机临床试验比较首次复发后首选第二次ASCT与持续药物治疗的结果，英国MRC X试验显示第二次ASCT是一种安全有效的治疗方法，PFS和OS均优于单纯化疗[37]；德国GMMG Ⅲ期ReLApsE试验显示，如果患者能按计划进行第二次ASCT，PFS和OS均优于Rd持续治疗[38]。因此，对于计划实施ASCT的患者，建议在初始诱导治疗后采集足够两次移植的造血干细胞以备复发后行第二次ASCT。

影响晚期ASCT和第二次ASCT实施与否的主要因素包括是否有足够的造血干细胞及复发后患者的状态。复发和第一次ASCT相隔时间在24个月以上、再次诱导后达CR及以上疗效、无获得性高危细胞遗传学及髓外浆细胞瘤的患者行第二次移植的疗效较好。

（三）我国首次复发MM患者的治疗推荐

首次复发后应先评估是否适合行ASCT，如一线接受过ASCT治疗，PFS时间在24个月以上，或一线治疗未接受ASCT，首次复发后评估适合ASCT且有足够的造血干细胞，再次诱导治疗达部分缓解（PR）及以上疗效者，可以考虑行第二次或晚期ASCT。ASCT前的再诱导治疗方案目前尚无前瞻性研究结果供参考，建议如复发距初次诱导有效的方案间隔6个月以上，可选择初次诱导有效的方案，也可选择与既往诱导治疗作用机制不同的方案进行再诱导（见下文）。无论是否选择移植，目前用于治疗RRMM患者的药物在美国FDA、国外各大指南及NMPA（中国国家药品监督管理局）的获批情况见表3。

**表3:** 目前中国获批治疗多发性骨髓瘤的新药情况和指南推荐

药物	FDA	ESMO[32]	IMWG[33]	NCCN[39]	MAYO[34]	NMPA
达雷妥尤单抗	NDMM；RRMM	NDMM；首次复发；多线复发	首次复发；多线复发	NDMM；RRMM	NDMM；首次复发；多线复发	NDMM；RRMM
卡非佐米	RRMM；NDMM（超说明书）	首次复发；多线复发	首次复发；多线复发	NDMM；RRMM	首次复发	RRMM
泊马度胺	RRMM	首次复发；多线复发	首次复发；多线复发	RRMM	首次复发	RRMM
Selinexor	RRMM	首次复发；多线复发	首次复发；多线复发	RRMM	多线复发	RRMM

注：FDA：美国食品药品监督管理局；ESMO：欧洲肿瘤内科学会；IMWG：国际骨髓瘤工作组；NCCN：美国国立综合癌症网络；MAYO：美国梅奥医学中心；NMPA：中国国家药品监督管理局；NDMM：初诊多发性骨髓瘤；RRMM：复发难治多发性骨髓瘤

1. 来那度胺耐药：对于来那度胺耐药的患者，结合我国目前新药的可及性，可选择单克隆抗体（达雷妥尤单抗）、新一代IMiDs（泊马度胺）、PIs（硼替佐米、卡非佐米）、其他作用机制药物（塞利尼索）（图1）。

**图1:**
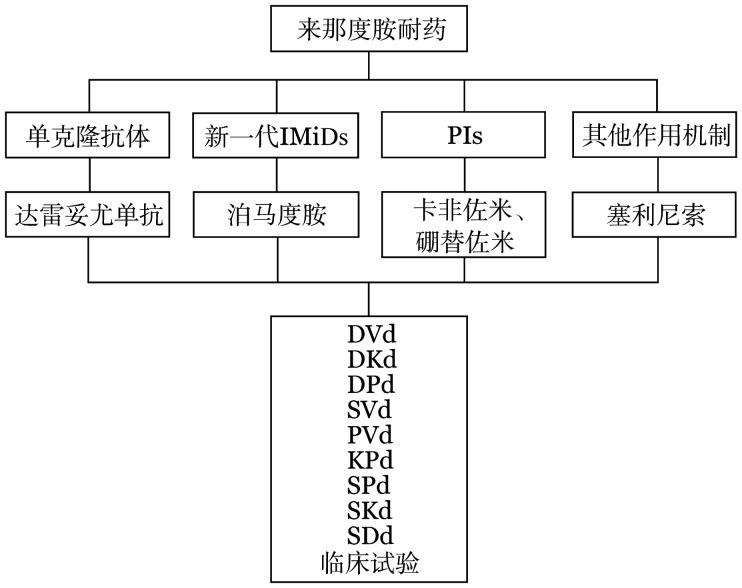
来那度胺耐药的首次复发多发性骨髓瘤患者的治疗选择 IMiDs：免疫调节剂；PIs：蛋白酶体抑制剂；D：达雷妥尤单抗；V：硼替佐米；d：地塞米松；K：卡非佐米；P：泊马度胺；S：塞利尼索

2. 硼替佐米耐药：对于硼替佐米耐药的患者，结合我国目前新药的可及性，药物选择有单克隆抗体（达雷妥尤单抗）、新一代PIs（卡非佐米）、IMiDs（来那度胺、泊马度胺）、其他作用机制药物（塞利尼索）（图2）。

**图2:**
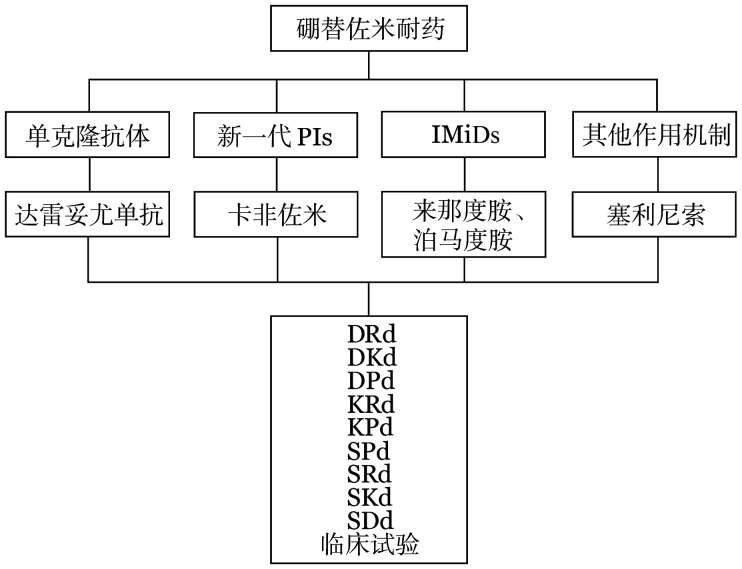
硼替佐米耐药的首次复发多发性骨髓瘤患者的治疗选择 PIs：蛋白酶抑制剂；IMiDs：免疫调节剂；D：达雷妥尤单抗；R：来那度胺；d：地塞米松；K：卡非佐米；P：泊马度胺；S：塞利尼索

3. 来那度胺和硼替佐米双重耐药：对于来那度胺和硼替佐米双重耐药的患者，结合我国目前新药获批的可及性，药物选择有单克隆抗体（达雷妥尤单抗）、新一代PIs（卡非佐米）、新一代IMiDs（泊马度胺）、其他作用机制药物（塞利尼索）（图3）。

**图3:**
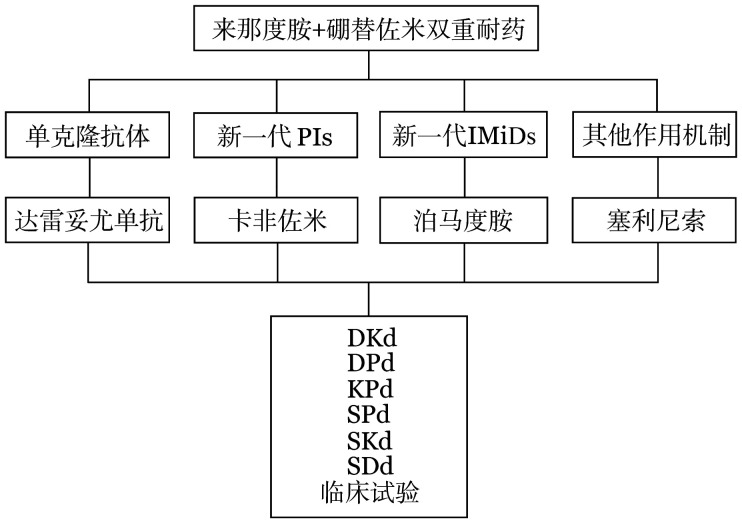
来那度胺和硼替佐米双重耐药首次复发多发性骨髓瘤患者的治疗选择 PIs：蛋白酶体抑制剂；IMiDs：免疫调节剂；D：达雷妥尤单抗；K：卡非佐米；d：地塞米松；P：泊马度胺；S：塞利尼索

4. 来那度胺、硼替佐米和达雷妥尤单抗三重耐药：达雷妥尤单抗在我国已获批用于治疗不适合移植的初诊MM（NDMM）患者，首次复发可能会出现三重耐药情况（PIs、IMiDs和达雷妥尤单抗耐药）。三重耐药的患者在首次复发时可选择新一代的PIs（卡非佐米）、IMiDs（泊马度胺）和其他作用机制药物塞利尼索。目前尚无达雷妥尤单抗一线治疗后耐药的临床试验，结合专家共识及对达雷妥尤单抗耐药的多线RRMM患者的数据，并结合目前我国药物的可及性，进行如下推荐（图4）。

**图4:**
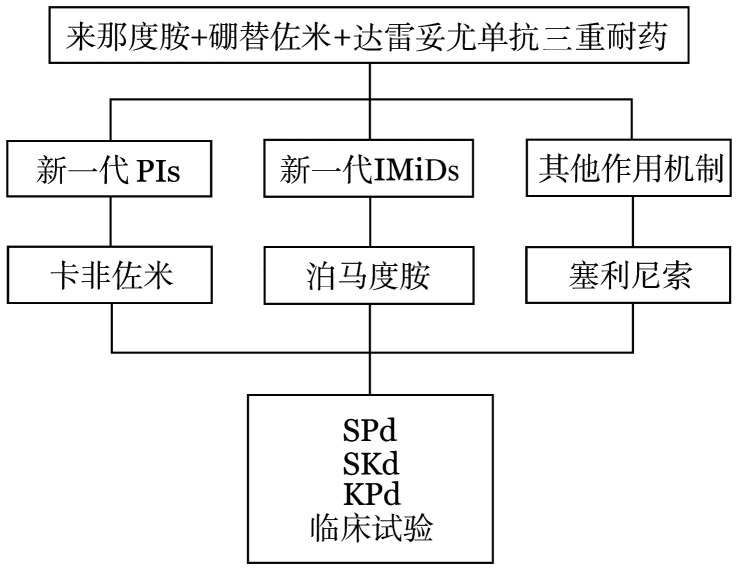
来那度胺、硼替佐米和达雷妥尤单抗三重耐药的首次复发多发性骨髓瘤患者的治疗选择 PIs：蛋白酶体抑制剂；IMiDs：免疫调节剂；S：塞利尼索；P：泊马度胺；d：地塞米松；K：卡非佐米

在临床实际选择方案时需注意以下问题：首次复发开始治疗前患者全血细胞明显减少，慎用有骨髓抑制作用的药物或需要调节剂量，如IMiDs、塞利尼索；首次复发后如果周围神经炎（1级伴疼痛或2级以上）明显，避免应用硼替佐米；严重心脏病慎用卡非佐米；严重感染慎用达雷妥尤单抗；对于高危患者，也可根据以上原则选择含有CD38单抗的四药联合方案。对于选择晚期或第二次ASCT的患者，移植后进入维持治疗，建议选用和首次复发前维持治疗作用机制不同或新一代作用机制的药物。对于不适合ASCT的患者，建议应用有效方案化疗8～9个疗程，随后进入维持治疗，维持治疗方案选择的原则同上。

5. 其他特殊复发情况的处理：

（1）髓外复发：对于髓外复发的MM患者无特别有效的方案，有条件者可进入如CAR-T细胞治疗的临床试验，或者选用如下方案：苯达莫司汀联合VTD、VRD或联合相应的新一代药物；塞利尼索联合Vd、Kd、Rd、Pd；细胞毒药物的多药联合，如新药联合DT-PACE方案；局部放疗等。髓外复发特别是血行播散型预后较差，中枢神经系统髓外浸润者预后更差，可通过血脑屏障的药物包括苯达莫司汀、泊马度胺、来那度胺和塞利尼索等可供选择。

（2）浆细胞白血病：以浆细胞白血病形式复发的患者（继发性浆细胞白血病）预后差，缺乏有效的治疗方案，可选用新的作用机制药物和（或）细胞毒药物的多药联合，如新药联合DT-PACE方案。但由于这类患者既往接受过多种药物治疗，骨髓功能均较差，选择有骨髓抑制作用的方案需特别注意。

（3）老年患者的治疗选择：对于老年患者，建议按照国际骨髓瘤工作组（IMWG）老年评估（GA）系统进行体能状态的评估。评分为Fit（良好）且符合ASCT条件的患者可进行ASCT，不符合ASCT条件的患者按上述推荐的方案进行治疗；评分为Intermediate-fitness（中等）的患者，可按照以上原则选择强度减低的三药联合，也可选择两药联合，如Pd、Kd、Vd、Rd、Darad、Sd等。评分为Frail（虚弱）的患者，建议在支持治疗基础上给予剂量调整的两药联合。

（4）高危年轻MM患者：如有合适供者，可考虑行异基因造血干细胞移植。
